# Model-Based PAT for Quality Management in Pharmaceuticals Freeze-Drying: State of the Art

**DOI:** 10.3389/fbioe.2017.00005

**Published:** 2017-02-07

**Authors:** Davide Fissore

**Affiliations:** ^1^Dipartimento di Scienza Applicata e Tecnologia, Politecnico di Torino, Torino, Italy

**Keywords:** freeze-drying, process analytical technology, control, optimization, design space, pharmaceuticals

## Abstract

Model-based process analytical technologies can be used for the in-line control and optimization of a pharmaceuticals freeze-drying process, as well as for the off-line design of the process, i.e., the identification of the optimal operating conditions. This paper aims at presenting the state of the art in this field, focusing, particularly, on three groups of systems, namely, those based on the temperature measurement (i.e., the soft sensor), on the chamber pressure measurement (i.e., the systems based on the test of pressure rise and of pressure decrease), and on the sublimation flux estimate (i.e., the tunable diode laser absorption spectroscopy and the valveless monitoring system). The application of these systems for in-line process optimization (e.g., using a model predictive control algorithm) and to get a true quality by design (e.g., through the off-line calculation of the design space of the process) is presented and discussed.

## Introduction

Freeze-drying is a process widely used in pharmaceuticals manufacturing, aiming to recover an active pharmaceutical ingredient from a liquid solution with the goal of increasing its stability over time and, thus, its shelf life (Fissore, 2013).

With respect to other drying processes, freeze-drying is generally the preferred technology in the pharmaceutical field, as the low temperature of the process avoids jeopardizing product characteristics (e.g., the pharmaceutical activity), being active pharmaceutical ingredients highly heat sensitive (Pikal and Dellerman, 1989; Carpenter et al., 1997; Franks, 1998; Leader et al., 2008).

In a freeze-drying process, the product is at first cooled at low temperature (e.g., −50°C) in such a way that the solvent (water, in most cases) freezes. Then, the pressure in the equipment where the process is carried out is decreased, in such a way that ice sublimation can occur (primary drying). During this stage, the temperature of the product is increased, and heat is continuously supplied to the product as ice sublimation is an endothermic process. As during the freezing stage not all the water leaves the product, but a small amount remains bound to product molecules, the drying process is usually not completed at the end of the primary drying stage. It is necessary, in fact, to further increase the temperature of the product to promote water desorption, in such a way that the desired amount of residual humidity is obtained in the final product (Mellor, 1978; Jennings, 1999; Oetjen and Haseley, 2004; Franks, 2007). Nowadays, the number of products requiring freeze-drying in the manufacturing stage is rapidly increasing, as a consequence of the ever-increasing number of molecules like proteins, peptides, vaccines, cell-based products, monoclonal antibodies, and oligonucleotides put on the market.

The freeze-drying process is generally carried out in a batch mode: the product is processed either in vials or in trays, loaded onto the shelves of the drying chamber. Product cooling, in the freezing stage, and heating, in the drying stages, is obtained through a technical fluid that flows into the shelves. The desired vacuum level is obtained using a vacuum pump and a condenser, where the vapor leaving the product is removed. In some cases, a controlled leakage of inert gas is used to achieve a better pressure control.

Despite the low values of pressure and temperature characterizing the process, the quality of the product can be jeopardized in case its temperature trespasses a threshold value, which is a characteristic of the product being processed. In case of an amorphous product, the limit temperature is related to the collapse of the dried cake, which can be responsible for product quality degradation, higher residual moisture, and longer drying time (Bellows and King, 1972; Tsourouflis et al., 1976; Adams and Irons, 1993; Pikal, 1994; Franks, 1998; Wang et al., 2004). When processing a crystalline product, the limit value is the melting temperature, to avoid the formation of a liquid product. Besides, the limit temperature can be related also to the thermal stability of the molecule processed. A further constraint that should be considered is connected to the sublimation flux: on one side, it has to be compatible with the capacity of the condenser, on the other side the occurrence of choking flow in the duct connecting the chamber to the condenser has to be avoided, as it implies the loss of pressure control in the chamber (Searles, 2004; Nail and Searles, 2008; Patel et al., 2010). Finally, as the process can be highly time consuming, and the identification of the operating conditions that allow minimizing the duration of the process and, especially, of the primary drying stage should be an important concern. It is therefore necessary to set adequate values of chamber pressure and shelf temperature during the drying process aiming to fulfill the previously listed targets and constraints.

A trial and error approach is usually used to design a freeze-drying process, testing (experimentally) a certain set of operating conditions and, then, evaluating product quality at the end of the process. Clearly, by this way, the time required to design the process can be very high (as well as the cost), and a real optimization of the process cannot be assured. In this framework, a change of paradigm occurred in 2004 with the issue of the “Guidance for Industry PAT” by the US Food and Drug Administration. The central point of this document is that a pharmaceutical process must be designed and carried out in such a way that product quality is no longer tested at the end of the process, but it is built-in, or is by design. This result can be achieved by using suitable process analytical technology (PAT) tools, i.e., systems for designing, monitoring, and controlling a process with the goal of ensuring final product quality.

Beside physical sensors, mathematical modeling can play a significant role in the development of PAT tools. It is well known that a mathematical model is a simplified representation of a system (process) that can be used to predict the behavior of a system under a set of conditions and, possibly, to enhance the scientific understanding. The existing knowledge about the system, the objective of the study, and the data available drive the formulation of the model. The level of oversight of the model should be commensurate with the level of risk associated with its use and, particularly, with its contribution in assuring the quality of the product. According to the Endorsed Guide for Q8/Q9/Q10 Implementation issued by the ICH (International Council for Harmonisation of Technical Requirements for Pharmaceuticals for Human Use), it is possible to distinguish between:

– low-impact models, typically used to support product and/or process development;
– medium-impact models, generally used to assure product quality (with other indicators);
– high-impact models, whose predictions are a significant indicator of product quality.

As it will be shown in the following, low-impact models are at the basis of various PATs used for both the off-line and for the in-line process design/optimization. The in-line optimization can be achieved using a control system as well as algorithms that calculate the values of the operating conditions (the temperature of the heating fluid and the pressure in the drying chamber) using a model of the process and the measurement of some process variables. The off-line design of the process requires the calculation, using a model of the process, of the design space, defined, by the ICH Q8 Pharmaceutical Development Guideline, as “the multidimensional combination of input variables and process parameters that have been demonstrated to provide assurance of quality.”

The goal of this paper is to discuss the state of the art in the field of the model-based PATs for quality management in pharmaceuticals freeze-drying. According to the previously listed targets and constraints of a freeze-drying process, such techniques can be used:

– to monitor/estimate product temperature, thus evidencing if the threshold value is trespassed;
– to monitor/estimate the residual amount of ice, thus identifying the ending point of the main drying stage;
– to estimate the parameters of the selected mathematical model, in such a way that the model can be used for off-line optimization of the process (i.e., the identification of the values of chamber pressure and heating shelf temperature that allow minimizing the drying time, beside fulfilling the constraint on the threshold temperature);
– to optimize the in-line process.

At first, model-based PAT tools based on temperature measurement will be introduced, pointing out how they can be used for in-line and off-line process development. Then, the focus will be on those PAT tools based on the pressure measurement and, finally, on those based on the estimation of the sublimation flux. Finally, the industrial application of these tools will be discussed in the Section “Conclusion.”

## Model-Based PATs Based on the Measurement of Product Temperature

Product temperature can be directly measured using either a thermocouple or a resistance thermal detector. With the goal of estimating also other important variables, e.g., the sublimation flux and, thus, the residual amount of ice in the product, the measurement of product temperature can be used with a mathematical model to get an observer.

One of the elements of the observer is a mathematical model of the process, generally constituted by a set of non-linear differential equations relating the state variables (**x**) and the manipulated input (*u*) to the variation of the state variables vs. time (*t*):

(1) $$\frac{d\text{x}}{\mathrm{dt}}=\text{f}\left(\text{x},u\right)$$

and by an equation relating the output (measured) variable (*y*) to the state variables and the manipulated input:

(2) y=h(x,u)

The functions **f** and *h* are obtained from model equations of the process. The measured values of the output variables can not be coincident with the values calculated using the model, due to model approximations and the uncertainty of the experimental measurement. As a consequence, the difference between calculated and measured values of the output variables can be used to “correct” the model, thus obtaining the equations of the observer:

(3) $$\frac{d\hat{\text{x}}}{\mathrm{dt}}=\text{f}\left(\hat{\text{x}},u\right)+\text{K}\left(\hat{y}-y\right)$$

(4) $$\hat{y}=h\left(\hat{\text{x}},u\right)$$

The model of the process is thus used to calculate the evolution of the (estimated) state variables ($\hat{\text{x}}$) through the vectorial function **f** and the difference between the estimated ($\hat{y}$) and the measured (*y*) values of the output variables, multiplied by the parameter **K**, the “gain” of the observer, that can be calculated using various algorithms, with the goal of obtaining the convergence of the observer, i.e., of driving the estimation error ($\hat{y}$ − *y*) to zero. The user can start the calculations with a first guess of the state variables $\left(\hat{\text{x}}\left(0\right)\right)$ and, then, the measurement of the output variables is used to “refine” model calculations: by this way, a reliable estimate of the state variables is obtained through mathematical simulation.

In the algorithm of the observer, a simple model can be used, as the experimental measure is used in the observer algorithm. In all the realizations of the observer (for freeze-drying monitoring) appeared in the literature, the model of Velardi and Barresi (2008) is used. It assumes that radial gradients of temperature and compositions are negligible, both in the frozen and in the dried portion of the product, and that heat accumulation in the frozen product is negligible and, thus, all the heat entering the frozen product is used for ice sublimation. The heat flux to the product (*J_q_*) and the sublimation flux (*J_w_*) are thus related through the following equation:

(5) $$J_{q}=\Delta H_{s}J_{w}$$

where Δ*H_s_* is the heat of sublimation. The heat flux to the product is proportional to the temperature difference between the heating fluid flowing in the shelf (*T*_fluid_) and the product at the bottom of the container (*T*_B_):

(6) $$J_{q}=K_{v}\left(T_{\text{fluid}}-T_{\text{B}}\right)$$

*K_v_* is the overall heat transfer coefficient between the heating fluid and the product in the container: it depends on the chamber pressure, on the type of vial and freeze-dryer used, and on the position of the vial over the shelf as all these parameters may affect the heat transfer to the product. The sublimation flux is proportional to the difference between the water vapor partial pressure at the interface of sublimation (*p_w,i_*) and in the drying chamber (*p_w,c_*):

(7) $$J_{w}=\frac{1}{R_{p}}\left(\;p_{w,i}-p_{w,c}\right)$$

*R_p_* is the resistance of the dried cake to vapor flux, and it depends on the type of product, as well as on the freezing protocol, and on the thickness of the dried layer (*L*_dried_), generally, in a non-linear way:

(8) $$R_{p}=R_{p,0}+\frac{A_{R_{p}}L_{\text{dried}}}{1+B_{R_{p}}L_{\text{dried}}}$$

The second equation of the model (beside Eq. 5) is the mass balance for the frozen layer:

(9) $$\frac{dL_{\text{frozen}}}{\mathrm{dt}}=-\frac{1}{\rho_{\text{frozen}}-\rho_{\text{dried}}}J_{w}$$

where ρ_frozen_ and ρ_dried_ are, respectively, the density of the frozen and of the dried product, and *L*_frozen_ is the thickness of the frozen layer.

Velardi et al. (2009, 2010) proposed two different observers for the primary drying stage, using the extended Kalman filter and the high gain technique to calculate the gain **K**. Although the extended Kalman filter (Kalman, 1960) was originally proposed to cope with the experimental measurement uncertainty (and not with model approximations), such algorithm was demonstrated to provide accurate estimates of the desired variables due to the accuracy of the model used. The original algorithm of Velardi et al. (2009) was then modified by Bosca and Fissore (2011) to account for the non-linear dependence of the resistance of the dried product on its thickness. Further improvements were introduced by Bosca et al. (2013a,b, 2014) to reduce the number of estimated variables and, thus, to improve the robustness of the system, as well as to get reliable estimates of the model parameters required by the observer (Bosca et al., 2015a). In the last formulation, the equations of the observer are the following:

(10) $$\left(\begin{array}{c} \frac{d{\hat{T}}_{i}}{\mathrm{dt}}\\ \frac{d{\hat{K}}_{v}}{\mathrm{dt}} \end{array}\right)=\left(\begin{array}{c} \text{f}\left({\hat{T}}_{i},{\hat{K}}_{v},T_{\text{fluid}}\right)\\ 0 \end{array}\right)+\text{K}\left({\hat{T}}_{\text{B}}-T_{\text{B}}\right)$$

(11) $${\hat{T}}_{\text{B}}=h\left({\hat{T}}_{i},{\hat{K}}_{v},T_{\mathrm{fluid}}\right)$$

where ${\hat{T}}_{i}$ is the estimate of product temperature at the interface of sublimation, ${\hat{T}}_{\text{B}}$ and *T*_B_ are, respectively, the estimated and the measured values of product temperature at the bottom of the vial, ${\hat{K}}_{v}$ is the estimate of the heat transfer coefficient between the heating fluid and the product, and **f** and *h* are non-linear functions obtained from the model of Velardi and Barresi (2008) previously described. Considering Eq. 10, it appears that the observer is used not only to estimate the state of the product but also the model parameter *K_v_*. With respect to the other parameter *R_p_*, the values of $B_{R_{p}}$ and *R_p_*,_0_ are kept constant and equal to their first estimates, while the parameter $A_{R_{p}}$ is calculated from Eq. 5, using Eqs 6–8, thus obtaining:

(12) $$A_{R_{p}}=\frac{\Delta H_{s}\left(1+B_{R_{p}}L_{\text{dried}}\right)\left(p_{w,i}-p_{w,c}\right)}{K_{v}\left(T_{\text{fluid}}-T_{B}\right)L_{\text{dried}}}-\frac{R_{p,0}+R_{p,0}B_{R_{p}}L_{\text{dried}}}{L_{\text{dried}}}$$

The first estimate of the parameters *R_p_*_,0_, $A_{R_{p}}$, and $B_{R_{p}}$ are obtained using the measurement of product temperature in the freezing stage to calculate the axial distribution of the ice crystal diameters [using the model of Nakagawa et al. (2007)] and, then, the estimate of the resistance of the dried product vs. *L*_dried_ is obtained using the method of Kuu et al. (2013). With respect to the first estimate of *K_v_*, a least-square problem is solved considering the measurement of product temperature in the first 30 min after the onset of the primary drying stage, and looking for the best fit between measured and calculated values of product temperature.

With respect to the temperature measurement, it should be highlighted that the insertion of a thermocouple in the vial containing the product does not affect importantly the nucleation of the ice crystals (and, thus, the structure of the dried cake), at least when the process is carried out in non-good manufacturing practice conditions (Bosca et al., 2013c) and, thus, the observer can be safely used for cycle development. Besides, it should be considered that the measurement of product temperature is not reliable in the final part of the primary drying stage. In fact, before the ending of the ice sublimation, an abrupt increase of the temperature value measured by the thermocouple is observed, with a slope different from that of the first part of the drying stage, even in case the heating fluid temperature is not modified. This can be due to various (and not yet fully understood) reasons, e.g., the loss of contact between the tip of the thermocouple and the product, or the fact that the sublimation front advances past the tip of the thermocouple. In any case, in the last part of the primary drying stage, the temperature measurement is not reliable and, thus, the observer calculations must be stopped. The evolution of the product can be estimated till the end of the ice sublimation using the model of the process, and the parameters estimated by the observer, taking advantage of the fast convergence of the observer. As an alternative, it is possible to measure the temperature of the product with plasma sputtered thermocouples, obtained through low-pressure plasma processes (Grassini et al., 2013; Parvis et al., 2014; Oddone et al., 2015): by this way, a non-invasive monitoring of product temperature can be achieved.

**Figure 1:**
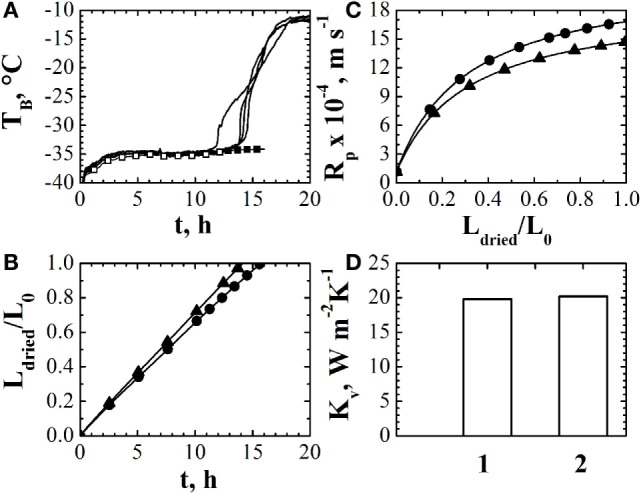
**(A)** Comparison between the experimentally measured values of product temperature obtained through two thermocouples inserted into different vials of the batch (−) estimates of the observer (◦) and of the mathematical model (•). **(B)** Estimated values of the dried cake thickness, given as ratio with the initial product thickness, using different temperature measurements. **(C)** Estimated values of the resistance if the dried cake to vapor flow using different temperature measurements. **(D)** Estimated values of the heat transfer coefficient using different temperature measurements. Data refer to the drying of a 5% by weight sucrose solution processed at *T*_fluid_ = −20°C and *P_c_* = 10 Pa in glass tubing vials ISO 83623-1 8R, with a filling volume of 2 mL arranged directly on the shelf according to an hexagonal array. Data taken from Bosca et al. (2015b); reprinted with permission from Bosca et al. (2015b). Copyright 2015 American Chemical Society.

An example of the results that can be obtained using an observer (and the mathematical model when the temperature measurement is no longer reliable) is shown in Figure 1. The temperature measurements used by the observer are shown in Figure 1A: generally, in a freeze-drying process, various thermocouples, placed into different vials, can be used and, thus, the observer can be run using different temperature measurements. By this way, it is possible to get a mean value of the desired variables (heat transfer coefficient, dried cake resistance, drying time, etc.) and the uncertainty range. With respect to the temperature measurement, as shown in Figure 1A, there are no differences in the estimated values when different measures are used. Up to about 12 h, the temperature measurement is used by the observer, and the comparison between the estimated and the measured values of product temperature can be used to point out the accuracy of the observer estimates. After 12 h, the mathematical model, with the value of the parameters estimated by the observer, is used to predict the evolution of the temperature of the product till the end of the primary drying stage. The evolution of the thickness of the dried layer is shown in Figure 1B, for the two temperature measurements considered in the experiment: it appears that after about 16 h the primary drying stage is completed, as confirmed also by the temperature measurement. In fact, at the end of the primary drying stage, product temperature approaches the value of the heating fluid (or a higher value, in case radiation from chamber walls plays an important role) as the heat arriving to the product is no longer used for ice sublimation, as confirmed by Figure 1A. The values of the parameters *R_p_* vs. *L*_dried_ and *K_v_* are shown in Figures 1C,D, respectively.

**Figure 2:**
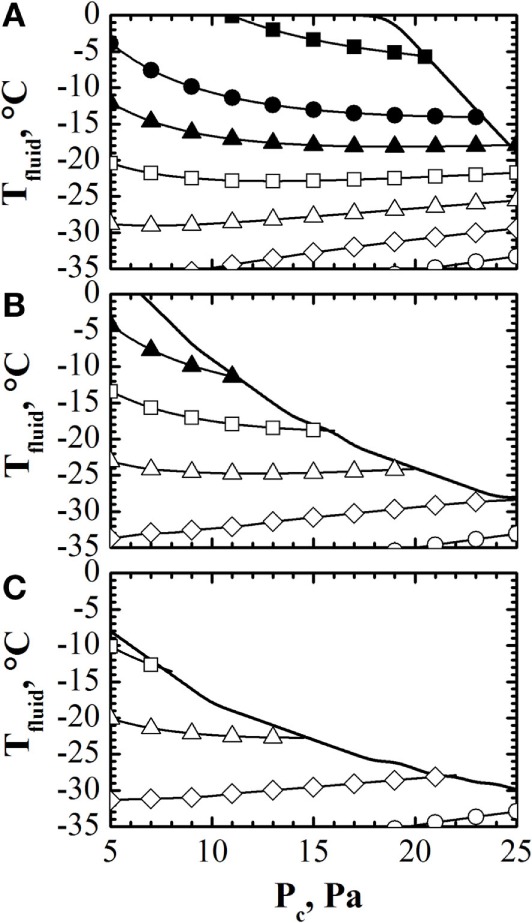
**Design space (solid line) calculated at the onset [panel (A)], in the middle [panel (B)] and at the end [panel (C)] of the primary drying stage, with contour plot of the sublimation flux (■ = 0.4, • = 0.30, ▴ = 0.25, □ = 0.2, Δ = 0.1, ♢ = 0.05, ◦ = 0.01 kg h^−1^ m^−2^) vs. *T*_fluid_ and *P_c_***. Data refer to the drying of a 10% by weight sucrose solution (*R_p_*_,0_ = 1.1 × 10^4^ m s^−1^, *A_Rp_* = 2.7 × 10^8^ s^−1^, *B_Rp_* = 2.5 × 10^3^ m^−1^) processed in glass tubing vials ISO 83623-1 2R with an initial filling height of 10 mm. Data taken from Fissore et al. (2011a).

Once model parameters have been estimated using the observer, it is possible to calculate the design space and, thus, to optimize off-line the process, using the model of the process. The target of the calculation is to evaluate the values of temperature of the heating fluid and pressure in the drying chamber (*P_c_*) that allow fulfilling the constraint on product temperature (and that on the sublimation flux, although this constraint is less demanding, at least in lab-scale units, where the sublimation flux is lower) and minimizing the duration of the primary drying stage. Giordano et al. (2011) proposed a simple algorithm, based on the model of Velardi and Barresi (2008), to calculate the design space of the process, considering to use the same values of *T*_fluid_ and *P_c_* throughout all the primary drying stage. The algorithm was improved by Fissore et al. (2011a), considering that the design space can change during the primary drying stage, as a consequence of the variation of the resistance of the dried product, and, thus, the cycle can be further optimized by taking into account this issue. An example of these calculations is shown in Figure 2: with these diagrams, it is possible to calculate the optimal values of *T*_fluid_ and *P_c_* that, at each time instant of the primary drying stage, allows maximizing the sublimation flux, considering the previously listed constraints of the process.

The observer can be used also to optimize in-line the process. Various tools were proposed to this purpose. Fissore et al. (2008) proposed a simple feedback controller, based on the proportional integral (PI) algorithm, that manipulates the temperature of the heating fluid on the basis of the difference between the measured value of product temperature and the target (limit) value. The tuning of the controller is performed minimizing the integral of the square error in the prediction horizon, i.e., in the time interval between the measurement of the state of the system and the ending point of the primary drying stage. The observer is used to get the estimates of product temperature when the reliable measurements are no longer available, as well as to get the values of model parameters required to simulate product dynamics in the tuning stage of the controller.

A different algorithm was proposed by Fissore (2015) based on the application of the fuzzy logic. In this case, the control system can manipulate both the temperature of the heating fluid and the pressure in the drying chamber with the goal of keeping product temperature as close as possible to the limit value, without trespassing it. Mathematical modeling is used just in the stage of control system design, aiming to define a unique set of fuzzy rules that can be used for a wide range of product. The observer, in this case, is used just to get an estimate of product temperature when reliable measurements are no longer obtained by the temperature probe.

**Figure 3:**
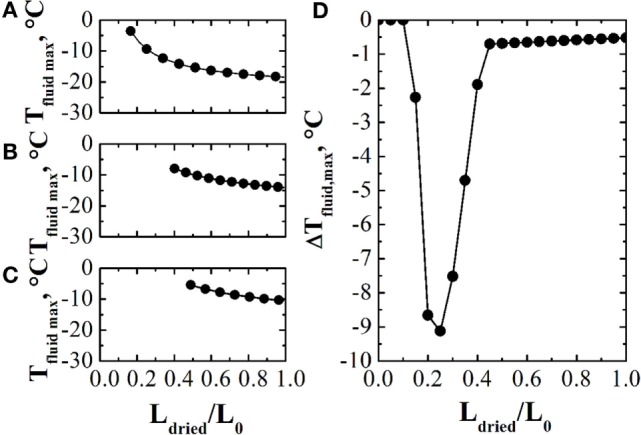
**Panels (A–C): design spaces, given as maximum allowed value of *T*_fluid_ vs. the thickness of the dried product (normalized through the initial product thickness), at three different instants during the primary drying stage**. Panel **(D)**: values of the difference between the actual product temperature and the limit one obtained using the design space based control system. Data refer to the drying of a 5% by weight sucrose solution, processed at *P_c_* = 5 Pa in glass tubing vials ISO 83623-1 2R with an initial filling height of 10 mm. Data taken from Bosca et al. (2013a).

Bosca et al. (2013b) proposed a method for the in-line optimization of the freeze-drying process based on the calculation of the design space of the process. Briefly, every prespecified time interval, e.g., 1 h, the design space of the primary drying stage is calculated using the estimates provided by the observer, and the temperature of the heating fluid is modified accordingly (chamber pressure is not optimized). Obviously, at the onset of the primary drying stage, the accuracy of the observer estimates can be poor and, for this reason, it is necessary to repeat the calculations as the process goes on, thus modifying *T*_fluid_ accordingly. An example of these calculations is shown in Figure 3, where in Figures 3A–C three design spaces, calculated at three different instants of the primary drying stage, are shown, while Figure 3D shows the performance of the system, given as the difference between the actual product temperature and the limit one, evidencing that, a part from the beginning of the primary drying stage, the temperature of the product rapidly approaches the limit value, without trespassing it.

## Model-Based PATs Based on the Measurement of Chamber Pressure

The measurement of chamber pressure during the pressure rise test is at the basis of various model-based PATs.

During the pressure rise test, the drying chamber is isolated from the condenser, closing the valve usually located in the duct connecting the chamber and the condenser. Chamber pressure increases due to the accumulation of water vapor and, at the same time, product temperature increases due to the fact the heating system is not stopped during the test: for this reason, the duration of the test is generally limited to 10–30 s. Once the measure of chamber pressure is available, a mathematical model is used to calculate the evolution of chamber pressure during the test: the parameters of the model [e.g., *K_v_* and *R_p_* in case the model of Velardi and Barresi (2008), is used] and the state of the product (i.e., its temperature and the residual amount of ice) are retrieved looking for the best fit between calculated and measured values of chamber pressure during the test. Such approach can thus be used for process monitoring, as it provides estimate of product temperature and residual amount of ice, and for process optimization, as the estimates of model parameters may allow the use of a mathematical model for off-line or in-line optimization, as it will be discussed in the following.

Various methods were proposed in the literature to get this result: they differ on the model equations used to calculate the pressure rise and, thus, on the number and type of the parameters calculated, although in call cases one of the parameters is the heat transfer coefficient, used to model the heat flux to the product, and the other is a coefficient used to model the mass transfer from the interface of sublimation to the drying chamber. The first algorithm, in chronological order, is the manometric temperature measurement (MTM), proposed by Milton et al. (1997), followed by the dynamic pressure rise proposed by Liapis and Sadikoglu (1998), by the pressure rise analysis of Chouvenc et al. (2004), and, finally, by the dynamic parameters estimation (DPE) proposed by Velardi et al. (2008), whose original algorithm was modified by Fissore et al. (2011b), aiming to improve the robustness of the algorithm (the new algorithm was labeled as DPE+), and by Pisano et al. (2011a), to account for the contribution of radiation to product heating.

When considering the pressure rise test to monitor the freeze-drying process, there are various issues that have to be considered:

- When a pressure rise test is carried out, it is possible to get the desired pieces of information (model parameters and state of the product) only for the time instant when the test is performed. In order to monitor the evolution of the product during the primary drying stage, it is thus necessary to repeat the test every prespecified time interval (e.g., every 30 min).
- During the pressure rise test, product temperature increases: it is thus necessary to account for this temperature rise when designing the process, and to modify the operating conditions in such a way that product temperature is well below the limit value during the primary drying stage, and it approaches the limit value when the test is carried out.
- When the pressure rise test is used for process monitoring, it is not possible to account for the non-uniformity of the batch. As a consequence of different heating mechanisms (e.g., radiation from chamber walls, that affect the dynamics of the product in the vials of the first rows), of pressure gradients in the chamber (Rasetto et al., 2010), of the non-uniform nucleation rate, the evolution of the product (i.e., the temperature and the residual amount of ice) is not the same in the various vials of the batch. Unfortunately, when the pressure rise test is used, “mean” values of model parameters of product temperature and of residual ice content are obtained, as the batch is assumed to be homogeneous. On the contrary, when using the temperature measurement for process monitoring, it is possible to account for batch non-uniformity placing thermocouples in different vials of the batch.
- Similar to the temperature measurement, also when the pressure rise test is used to monitor the process it is not possible to get accurate estimates of model parameters and of the state of the product in the last part of the primary drying stage. This is due to various reasons, as pointed out by Fissore et al. (2011b): one of the most important ones is the non-uniformity of the batch, as those vials receiving heat also by radiation from the chamber walls complete the drying before the others and, thus, at a certain moment, the number of vial where ice sublimation occurs changes, but this is not accounted for in any algorithm.
- Both product state and model parameters are obtained looking for the best fit between the calculated and the measured values of chamber pressure during the test. The solution of the least-square problem can thus impair the accuracy of the estimates obtained through this method, as pointed out by Bosca et al. (2016).

**Figure 4:**
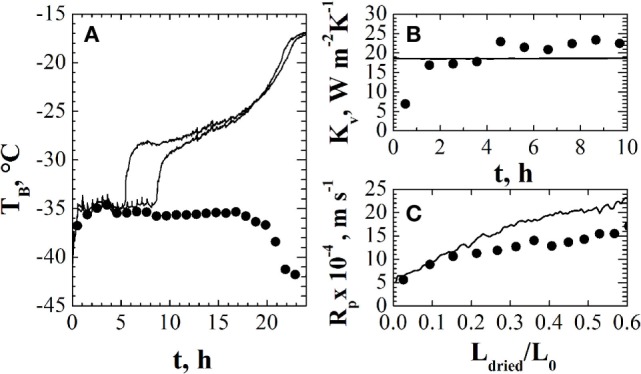
**Panel (A): comparison between product temperature measured by thermocouples (—) and estimated by DPE + algorithm (•)**. Panels **(B,C)**: comparison between the heat transfer coefficient [panel **(B)**] and the mass transfer resistance as a function of cake thickness [panel **(C)**] estimated by the soft sensor (—) and by DPE + algorithm (•). Data refer to the freeze-drying of a 5% (w/w) sucrose aqueous solution (*P_c_* = 10 Pa) in glass tubing vials ISO 83623-1 8R, with a filling volume of 2 mL arranged directly on the shelf according to a hexagonal array. Data taken from Bosca and Fissore (2014).

Figure 4 shows an example of the estimates obtained using one of the pressure rise test-based methods, namely, the DPE + algorithm. In Figure 4A, the estimates of product temperature are compared with the values measured using thermocouples, evidencing that rather good estimates of this variable are obtained, at least in the first part of the primary drying stage: in fact, after about 18 h from the onset of the primary drying, the estimates of product temperature starts decreasing, while it is expected that product temperature does not change until the ending of the ice sublimation (occurring after about 22 h from the onset of the primary drying). With respect to model parameters *K_v_* and *R_p_*, shown in Figures 4B,C, respectively, their values are compared to those obtained using the observer. It is possible to point out that different values are obtained using the two methods, about ±10/15% around the mean values (a value that can be considered acceptable in model parameters estimation for the freeze-drying process), but in both cases, the estimate of product temperature is correct. It has to be highlighted that in this test the edge vials have been replaced by empty vials, aiming to shield the batch from radiation effects and, thus, the non-uniformity of the batch is significantly decreased and the “mean” values estimated by DPE + algorithm are effectively representative of the real values of the vials of the batch.

The estimates of model parameters and product state obtained through the pressure rise test-based algorithm can be used in the framework of an in-line control system, aiming to identify the optimal values of the operating parameters (*T*_fluid_ and *P_c_*) in such a way that product temperature is as close as possible to the limit value, without trespassing it, and drying time is minimized. A first example of these systems is the Smart Freeze-Dryer by Tang et al. (2005): it is an “expert” system that manipulates *T*_fluid_ and *P_c_* using some empirical rules and the pressure rise test, with the MTM algorithm, as monitoring system. Such approach has no predictive capacity, i.e., it cannot determine the optimal values of the operating conditions considering the evolution of the system as a consequence of the selected values, and, thus, an effective process optimization cannot be achieved (Gieseler et al., 2007a). Pisano et al. (2010) proposed a different system, where the pressure rise test, and the DPE algorithm, is used to monitor the state of the product, but where a mathematical model is used to calculate the optimal heating policy (at constant chamber pressure). Two different algorithms are proposed: one is based on a classic feedback controller, where the parameters of the controller are calculated looking for the minimization of the error (difference between product temperature and the target value) along the prediction horizon, while the other is a model-based algorithm. In both cases, the system has a predictive capacity and, thus, it can prevent the occurrence of product temperature overshoots; besides, product temperature rise occurring during the pressure rise test is accounted for, and modeling errors are taken into account through a sliding horizon approach. Finally, Pisano et al. (2011b) proposed the use of advanced methods, based on the model predictive control technique, to optimize the primary drying stage, manipulating both the temperature of the heating fluid and the pressure in the drying chamber. This algorithm provides an implicit non-linear feedback, where modeling errors are directly embedded in the control actions calculation. By this way, both modeling approximations and parameter uncertainty can be compensated, and various process and operational constraints can be easily accounted for.

With the goal of overcoming the problem of the product temperature rise during the pressure rise test, Pisano et al. (2014) proposed to carry out a different test, called pressure decrease test, to monitor the state of the system. In this case, the drying chamber is no longer isolated from the condenser, but the pressure variation in the chamber is induced by closing the controlled leakage valve and, thus, chamber pressure decrease. Also, in this case, a mathematical model is used to calculate the values of chamber pressure during the test, and the state of the product (temperature and residual amount of ice) and the values of model parameters are retrieved looking for the best fit between calculated and measured values of chamber pressure. Different from the pressure rise test-based methods, this method requires a preliminary characterization of the freeze-dryer, aiming to correlate the flux in the duct connecting the chamber and the condenser to the pressure difference in the two environments. Moreover, it is necessary to measure not only the pressure in the chamber but also that in the condenser and, obviously, the method cannot be used in those freeze-driers where controlled leakage of inert gas is not used for pressure control.

## Model-Based PATs Based on the Estimate of Sublimation Flux

A third group of model-based PATs is based on the estimate of the sublimation flux. Different tools were proposed in the past to estimate the vapor flux leaving the drying chamber during the primary drying stage of a freeze-drying process. The working principle of these systems is quite simple:

1. once the vapor flow rate has been measured, this value is divided by the total surface area of the product, in such a way that the sublimation flux *J_w_* is calculated;
2. if product temperature (*T*_B_) is also measured, the heat transfer coefficient can be calculated using the following equation:
  (13) $$K_{v}=\frac{J_{w}\Delta H_{s}}{\left(T_{\text{fluid}}-T_{\text{B}}\right)A_{v}}$$
  where *A_v_* is the total sublimation area of the product;
3. with respect to the dried cake resistance, this parameter can be calculated using the following equation:
  (14) $$R_{p}=\frac{p_{w,i}-p_{w,c}}{J_{w}}$$

assuming that water vapor partial pressure in the drying chamber (*p_w,c_*) is equal to total chamber pressure, and calculating water vapor partial pressure at the interface of sublimation using one of the equations proposed to correlate this value to the temperature of the interface of sublimation [e.g., the equation proposed by Goff and Gratch (1946)], and assuming that the temperature at the interface of sublimation is equal to *T*_B_ (being axial gradient of temperature in the frozen product very small).

It appears that a system based on the estimate of the sublimation flux, like that based on the pressure rise test, is able to estimate “mean” values of model parameters, as the batch is assumed homogeneous (i.e., the sublimation flux is assumed to be the same in all the vials of the batch). Besides, in order to estimate *K_v_* and *R_p_*, it is necessary to measure also product temperature at the bottom of the vial. In case *T*_B_ is unknown, e.g., because it is not possible to measure product temperature, then it is required to carry out a preliminary investigation to get the value of *K_v_*. Then, during the primary drying stage the measure of *J_w_* can be used to estimate *T*_B_ using the following equation:

(15) $$T_{\text{B}}=T_{\text{fluid}}-\frac{J_{\text{w}}}{\Delta H_{s}K_{v}}$$

and once *T*_B_ is known, then *R_p_* can be calculated using Eq. 14. Finally, once model parameters are known, the design space can be calculated, and the process can be optimized off-line.

Various tools were proposed in the past to estimate the sublimation flux during the primary drying stage, e.g., the tunable diode laser absorption spectroscopy (TDLAS) (Gieseler et al., 2007b). This tool uses Doppler-shifted near-infrared absorption spectroscopy to measure the concentration of water vapor and the gas flow velocity in the duct connecting the chamber of the freeze-dryer and condenser. The vapor flux is then calculated on the basis of an estimate of the velocity profile in the duct. The effectiveness of this system for the estimation of *K_v_* and *R_p_* has been demonstrated (Kuu et al., 2009, 2011).

A different system is the valveless monitoring system proposed by Pisano et al. (2016): in this case, the rate of sublimation is estimated on the basis of the difference between the pressure in the drying chamber and in the condenser. Few experiments are required to get the parameter of the equation proposed to calculate the sublimation flux from the measure of such pressure drop. It has to be remarked that these calibration runs can be carried out using just the solvent, and not the drug, in any container, as the parameter required is not dependent on product and container characteristics. The effectiveness of the method for model parameters estimation and for cycle design was shown by Pisano et al. (2016).

## Conclusion

Although freeze-drying is a key step in pharmaceuticals manufacturing, and final product quality can be seriously impaired in case the operating conditions are not properly identified, even nowadays a trial and error approach is used to run the process, testing product quality in the final product. The pressure of regulatory agencies to provide evidences of the scientific rationale at the basis of the selected operating conditions and of its design space, and the pressure of the market, to reduce manufacturing costs and time, in particular for new products, are the main drivers for a change of approach. In this framework, model-based PATs are a unique tool for both process design, at R&D scale, and for process monitoring during manufacturing, as they allow going beyond the limits of the physical sensors through a careful use of mathematical modeling.

Considering that a freeze-drying process is generally designed at lab-scale, the temperature measurement, in the framework of the observer algorithm, is the unique system that allows for process monitoring and optimization taking into account the non-uniformity of the batch. The hardware required is very simple, as in almost every freeze-dryer one or more thermocouples are available, and, in every case, wireless temperature probes are on the market and can be used in the time of need.

The pressure rise test-based systems are effective at lab-scale, but it is required to verify if the test is feasible in the available freeze-dryer, and if chamber pressure can be monitored with an adequate sampling frequency. Besides, in order to improve the accuracy of the method, it is advisable to shield the batch from radiation from chamber walls/door, in such a way that the non-uniformity of the batch is minimized. The problem of temperature rise during the test, in case it is a critical issue, can be solved using the pressure decrease test. In industrial-scale units, the feasibility of the test is strictly related to the velocity of closure of the valve during the test.

With respect to the systems based on the estimate of the sublimation flux, the use of TDLAS is impaired by the fact that the calibration of the system can be tricky, and the installation of the device can be even impossible, depending on the way the condenser is connected to the drying chamber. On the other way, the valveless monitoring system can provide the same pieces of information, at a very low cost (just a second pressure sensor in the condenser), and with a very simple and fast calibration procedure.

Beside process monitoring, the control and optimization of the primary drying stage is of outmost importance as well. In this framework, if the goal is just to get an “adequate” cycle, then systems like LyoDriver and the Smart Freeze-Dryer, based on the pressure rise test, or the observer-based PI controller or the temperature-based fuzzy controller, are effective. Nevertheless, in my view, it is advisable to optimize off-line the process: in fact, by this way, it is possible to get the widest knowledge about the effect of the operating conditions on product dynamics (temperature and sublimation flux), evaluating also the robustness of the selected operating conditions.

At the end of this overview about the available model-based PATs, it should be clear to every freeze-drying practitioner that there is not a unique system that can be used to get the desired results: there are various tools that can be used depending on the hardware available, or that can be retrofitted, as well as on the goal of the work, i.e., cycle design in lab-scale units or process monitoring in industrial-scale freeze-dryers. These systems are available, and some of them are already on the market, and, thus, their effectiveness can be tested when a new product has to be freeze-dried.

## Author Contributions

The author confirms being the sole contributor of this work and approved it for publication.

## Conflict of Interest Statement

The author declares that the research was conducted in the absence of any commercial or financial relationships that could be construed as a potential conflict of interest.

## References

[B1] Adams G. D. J. Irons L. I. (1993). Some implications of structural collapse during freeze-drying using *Erwinia caratovora* l-asparaginase as a model. J. Chem. Technol. Biotechnol. 58, 71–76. 10.1002/jctb.280580110 7763938

[B2] Bellows R. J. King C. J. (1972). Freeze-drying of aqueous solutions: maximum allowable operating temperature. Cryobiology 9, 559–561. 10.1016/0011-2240(72)90179-4 4658017

[B3] Bosca S. Barresi A. A. Fissore D. (2013a). Fast freeze-drying cycle design and optimization using a PAT based on the measurement of product temperature. Eur. J. Pharm. Biopharm. 85, 253–262. 10.1016/j.ejpb.2013.04.008 23631849

[B4] Bosca S. Corbellini S. Barresi A. A. Fissore D. (2013b). Freeze- drying monitoring using a new process analytical technology: toward a “zero defect” process. Drying Technol. 31, 1744–1755. 10.1080/07373937.2013.807431

[B5] Bosca S. Barresi A. A. Fissore D. (2013c). Use of a soft-sensor for the fast estimation of dried cake resistance during a freeze-drying cycle. Int. J. Pharm. 451, 23–33. 10.1016/j.ijpharm.2013.04.046 23624086

[B6] Bosca S. Barresi A. A. Fissore D. (2014). Use of soft-sensors to monitor a pharmaceuticals freeze-drying process in vials. Pharm. Dev. Technol. 19, 148–159. 10.3109/10837450.2012.757786 23336717

[B7] Bosca S. Barresi A. A. Fissore D. (2015a). Design of a robust soft-sensor to monitor in-line a freeze-drying process. Drying Technol. 33, 1039–1050. 10.1080/07373937.2014.982250

[B8] Bosca S. Fissore D. Demichela M. (2015b). Risk-based design of a freeze-drying cycle for pharmaceuticals. Ind. Eng. Chem. Res. 51, 12928–12936. 10.1021/acs.iecr.5b03719

[B9] Bosca S. Barresi A. A. Fissore D. (2016). On the use of model-based tools to optimize in-line a pharmaceuticals freeze-drying process. Drying Technol. 34, 1831–1842. 10.1080/07373937.2016.1146755

[B10] Bosca S. Fissore D. (2011). Design and validation of an innovative for pharmaceuticals freeze-drying monitoring. Chem. Eng. Sci. 66, 5127–5136. 10.1016/j.ces.2011.07.008

[B11] Bosca S. Fissore D. (2014). Monitoring of a pharmaceuticals freeze-drying process by model-based process analytical technology tools. Chem. Eng. Tech. 37, 240–248. 10.1002/ceat.201300364

[B12] Carpenter J. F. Pikal M. J. Chang B. S. Randolph T. W. (1997). Rational design of stable lyophilized protein formulations: some practical advice. Pharm. Res. 14, 969–975. 10.1023/A:1012180707283 9279875

[B13] Chouvenc P. Vessot S. Andrieu J. Vacus P. (2004). Optimization of the freeze-drying cycle: a new model for pressure rise analysis. Drying Technol. 22, 1577–1601. 10.1081/DRT-200025605 16316065

[B14] Fissore D. (2013). “Freeze-drying of pharmaceuticals,” in Encyclopedia of Pharmaceutical Science and Technology, 4th Edn, ed. Swarbrick J. (London: CRC Press), 1723–1737.

[B15] Fissore D. (2015). On the design of a fuzzy logic-based control system for freeze-drying processes. J. Pharm. Sci. 105, 3562–3572. 10.1016/j.xphs.2016.08.018 27692619

[B16] Fissore D. Pisano R. Barresi A. A. (2011a). Advanced approach to build the design space for the primary drying of a pharmaceutical freeze-drying process. J. Pharm. Sci. 100, 4922–4933. 10.1002/jps.22668 21702051

[B17] Fissore D. Pisano R. Barresi A. A. (2011b). On the methods based on the Pressure Rise Test for monitoring a freeze-drying process. Drying Technol. 29, 73–90. 10.1080/07373937.2010.482715

[B18] Fissore D. Velardi S. A. Barresi A. A. (2008). In-line control of a freeze-drying process in vials. Drying Technol. 26, 685–694. 10.1080/07373930802046161

[B19] Franks F. (1998). Freeze-drying of bioproducts: putting principles into practice. Eur. J. Pharm. Biopharm. 45, 221–229. 10.1016/S0939-6411(98)00004-6 9653626

[B20] Franks F. (2007). Freeze-Drying of Pharmaceuticals and Biopharmaceuticals. Cambridge: Royal Society of Chemistry.

[B21] Gieseler H. Kramer T. Pikal M. J. (2007a). Use of manometric temperature measurement (MTM) and SMART™freeze dryer technology for development of an optimized freeze-drying cycle. J. Pharm. Sci. 96, 3402–3418. 10.1002/jps.20982 17853427

[B22] Gieseler H. Kessler W. J. Finson M. Davis S. J. Mulhall P. A. Bons V. (2007b). Evaluation of tunable diode laser absorption spectroscopy for in-process water vapor mass flux measurement during freeze drying. J. Pharm. Sci. 96, 1776–1793. 10.1002/jps.20827 17221854

[B23] Giordano A. Barresi A. A. Fissore D. (2011). On the use of mathematical models to build the design space for the primary drying phase of a pharmaceutical lyophilization process. J. Pharm. Sci. 100, 311–324. 10.1002/jps.22264 20575053

[B24] Goff J. A. Gratch S. (1946). “Low-pressure properties of water from – 160 to 212°F,” in Transactions of the American Society of Heating and Ventilating Engineers (New York: Proceedings of 52nd Annual Meeting of the American Society of Heating and Ventilating Engineers), 95–122.

[B25] Grassini S. Barresi A. Parvis M. (2013). Inert thermocouple with nanometric thickness for lyophilization monitoring. IEEE Trans. Instrum. Meas. 62, 1276–1283. 10.1109/TIM.2012.2223312

[B26] Jennings T. A. (1999). Lyophilization: Introduction and Basic Principles. Boca Raton: Interpharm/CRC Press.

[B27] Kalman R. E. (1960). A new approach to linear filtering and prediction. Trans. ASME J. Basic Eng. 82, 35–45. 10.1115/1.3662552

[B28] Kuu W. Y. Doty M. J. Rebbeck C. L. Hurst W. S. Cho Y. K. (2013). Gap-freezing approach for shortening the lyophilization cycle time of pharmaceutical formulations – demonstration of the concept. J. Pharm. Sci. 102, 2578–2588. 10.1002/jps.23610 23728733

[B29] Kuu W. Y. Nail S. L. Sacha G. (2009). Rapid determination of vial heat transfer parameters using tunable diode laser absorption spectroscopy (TDLAS) in response to step-changes in pressure set-point during freeze-drying. J. Pharm. Sci. 98, 1136–1154. 10.1002/jps.21478 18683861

[B30] Kuu W. Y. O’Bryan K. R. Hardwick L. M. Paul T. W. (2011). Product mass transfer resistance directly determined during freeze-drying cycle runs using tunable diode laser absorption spectroscopy (TDLAS) and pore diffusion model. Pharm. Dev. Technol. 16, 343–357. 10.3109/10837451003739263 20387998

[B31] Leader B. Baca Q. J. Golan D. E. (2008). Protein therapeutics: a summary and pharmacological classification. Nat. Rev. Drug Discov. 7, 21–39. 10.1038/nrd2399 18097458

[B32] Liapis A. I. Sadikoglu H. (1998). Dynamic pressure rise in the drying chamber as a remote sensing method for monitoring the temperature of the product during the primary drying stage of freeze-drying. Drying Technol. 16, 1153–1171. 10.1080/07373939808917458

[B33] Mellor J. D. (1978). Fundamentals of Freeze-Drying. London: Academic Press.

[B34] Milton N. Pikal M. J. Roy M. L. Nail S. L. (1997). Evaluation of manometric temperature measurement as a method of monitoring product temperature during lyophilisation. PDA J. Pharm. Sci. Technol. 51, 7–16. 9099059

[B35] Nail S. L. Searles J. A. (2008). Elements of quality by design in development and scale-up of freeze-dried parenterals. BioPharm Int. 21, 44–52.

[B36] Nakagawa K. Hottot A. Vessot S. Andrieu J. (2007). Modeling of freezing step during freeze-drying of drugs in vials. AIChE J. 53, 1362–1372. 10.1002/aic.11147

[B37] Oddone I. Fulginiti D. Barresi A. A. Grassini S. Pisano R. (2015). Non-invasive temperature monitoring in freeze drying: control of freezing as a case study. Drying Technol. 33, 1621–1630. 10.1080/07373937.2015.1040026

[B38] Oetjen G. W. Haseley P. (2004). Freeze-Drying, 2nd Edn Weinheim: Wiely-VHC.

[B39] Parvis M. Grassini S. Fulginiti D. Pisano R. Barresi A. A. (2014). “Sputtered thermocouple array for vial temperature mapping,” in Proceedings of IEEE International Instrumentation and Measurements Technology Conference “I2MTC 2014” (Montevideo, Uruguay), 1465–1470

[B40] Patel S. M. Swetaprovo C. Pikal M. J. (2010). Choked flow and importance of Mach I in freeze-drying process design. Chem. Eng. Sci. 65, 5716–5727. 10.1016/j.ces.2010.07.024

[B41] Pikal M. J. (1994). “Freeze-drying of proteins: process, formulation, and stability,” in Formulation and Delivery of Proteins and Peptides, eds Cleland J. L. Langer R. (Washington DC: American Chemical Society), 120–133.

[B42] Pikal M. J. Dellerman K. M. (1989). Stability testing of pharmaceuticals by high-sensitivity isothermal calorimetry at 25°C: cephalosporins in the solid and aqueous solution states. Int. J. Pharm. 50, 233–252. 10.1016/0378-5173(89)90127-0

[B43] Pisano R. Barresi A. A. Fissore D. (2011a). Innovation in monitoring food freeze-drying. Drying Technol. 29, 1920–1931. 10.1080/07373937.2011.596299

[B44] Pisano R. Fissore D. Barresi A. A. (2011b). Freeze-drying cycle optimization using model predictive control techniques. Ind. Eng. Chem. Res. 50, 7363–7379. 10.1021/ie101955a

[B45] Pisano R. Fissore D. Barresi A. A. (2014). A new method based on the regression of step response data for monitoring a freeze-drying cycle. J. Pharm. Sci. 103, 1756–1765. 10.1002/jps.23976 24756884

[B46] Pisano R. Fissore D. Barresi A. A. (2016). Noninvasive monitoring of a freeze-drying process for tert-butanol/water cosolvent-based formulations. Ind. Eng. Chem. Res. 55, 5670–5680. 10.1021/acs.iecr.5b04299

[B47] Pisano R. Fissore D. Velardi S. A. Barresi A. A. (2010). In-line optimization and control of an industrial freeze-drying process for pharmaceuticals. J. Pharm. Sci. 99, 4691–4709. 10.1002/jps.22166 20845466

[B48] Rasetto V. Marchisio D. L. Fissore D. Barresi A. A. (2010). On the use of a dual-scale model to improve understanding of a pharmaceutical freeze-drying process. J. Pharm. Sci. 99, 4337–4350. 10.1002/jps.22127 20301092

[B49] Searles J. (2004). Observation and implications of sonic water vapour flow during freeze-drying. Am. Pharmaceut. Rev. 7, 58–69.

[B50] Tang X. C. Nail S. L. Pikal M. J. (2005). Freeze-drying process design by manometric temperature measurement: design of a smart freeze-dryer. Pharm. Res. 22, 685–700. 10.1007/s11095-005-2501-2 15889467

[B51] Tsourouflis S. Flink J. M. Karel M. (1976). Loss of structure in freeze-dried carbohydrates solutions: effect of temperature, moisture content and composition. J. Sci. Food Agric. 27, 509–519. 10.1002/jsfa.2740270604

[B52] Velardi S. A. Barresi A. A. (2008). Development of simplified models for the freeze-drying process and investigation of the optimal operating conditions. Chem. Eng. Res. Des. 86, 9–22. 10.1016/j.cherd.2007.10.007

[B53] Velardi S. A. Hammouri H. Barresi A. A. (2009). In line monitoring of the primary drying phase of the freeze-drying process in vial by means of a Kalman filter based observer. Chem. Eng. Res. Des. 87, 1409–1419. 10.1016/j.cherd.2009.03.011

[B54] Velardi S. A. Hammouri H. Barresi A. A. (2010). Development of a high gain observer for inline monitoring of sublimation in vial freeze-drying. Drying Technol. 28, 256–268. 10.1080/07373930903530204

[B55] Velardi S. A. Rasetto V. Barresi A. A. (2008). Dynamic parameters estimation method: advanced manometric temperature measurement approach for freeze-drying monitoring of pharmaceutical. Ind. Eng. Chem. Res. 47, 8445–8457. 10.1021/ie7017433

[B56] Wang D. Q. Hey J. M. Nail S. L. (2004). Effect of collapse on the stability of freeze-dried recombinant factor VIII and α-amylase. J. Pharm. Sci. 93, 1253–1263. 10.1002/jps.20065 15067701

